# Supramolecular approaches to mediate chemical reactivity

**DOI:** 10.3762/bjoc.18.152

**Published:** 2022-10-14

**Authors:** Pablo Ballester, Qi-Qiang Wang, Carmine Gaeta

**Affiliations:** 1 Institute of Chemical Research of Catalonia (ICIQ), Avgda. Països Catalans 16, 43007 Tarragona, Spain; 2 Catalan Institution for Research and Advanced Studies (ICREA), Passeig Lluís Companys 23, 08010 Barcelona, Spain; 3 Beijing National Laboratory for Molecular Sciences, CAS Key Laboratory of Molecular Recognition and Function, Institute of Chemistry, Chinese Academy of Sciences, Beijing 100190, China; 4 University of Chinese Academy of Sciences, Beijing 100049, China; 5 Dipartimento di Chimica e Biologia “A. Zambelli”, Università di Salerno, I-84084 Fisciano, Salerno, Italy

Nature continuously inspires scientists to design novel prototypes of artificial systems with more and more advanced functions and properties [1–2]. In this regard, one of the greatest innovations was the discovery that small molecules can catalyze/mediate chemical reactions by a biomimetic approach [3–4]. Very recently, many efforts have been devoted to study supramolecular catalysis processes [5–14] in which macrocyclic hosts, self-assembled capsules and metallo-cages were employed as catalysts or nanocontainers.

The primary characteristic of supramolecular catalysis is that the general modes of activation based on intermolecular interactions can operate on substrates in a selective way, and in confined environment, like the active site of natural enzymes [5–14]. As a result, molecular recognition of the substrate(s) and potentially the transition state is essential in supramolecular catalysis.

Supramolecular catalysis finds inspiration in natural enzymes, which show catalytic features such as substrates and products selectivity, efficiency, geometric control, and acceleration of chemical reactivity [1]. If reactants are confined in the restricted space provided by an enzyme binding pocket, the increase in local concentration, due to the proximity effect, the stabilization of intermediates and transition states cause the acceleration of the reaction. Thus, learning from natural enzymes, novel supramolecular catalysts were designed that showed substrate selectivity, turnover, regioselectivity, and stereoselectivity [5–14].

Supramolecular architectures with an internal cavity are potential candidates to work as supramolecular catalysts [5–14]. The first supramolecular catalysts were based on covalent hosts, typically cyclodextrin macrocycles [1], which had an interior hydrophobic cavity decorated with catalytically useful functional groups. Recently, increased emphasis has been placed on catalysis that takes use of noncovalent hosts, including self-assembled capsules [5–6,8–9]. The capsules feature sizeable internal hydrophobic cavities that can accommodate large substrates and even allow bimolecular reactions to take place. In addition, they may ensure catalytic turnover. Transition states and reaction intermediates may also be stabilized in the cavities of the capsules by means of intermolecular interactions. The most intriguing aspect of catalysis in confined spaces [5–14] is that the reactions can take place through unusual mechanisms. This is mainly due to the conformational control of the substrates, steric constrictions, stabilization of species, and solvent exclusion phenomena occurring in the molecular containers isolated spaces [6]. Consequently, the classical rules of organic reactivity are often violated [6,11].

Taking into account the above considerations, we organized this thematic issue focused on showcasing innovative research regarding supramolecular catalysis using macrocyclic and acyclic hosts, as well as other molecular architectures such as self-assembled capsules and metallocages.

In their contribution, Secchi and Cera [15] reported the synthesis of diphosphine gold(I) calix[6]arene complexes whose geometry, in low-polarity solvents, is controlled by the 1,2,3-alternate conformation of the calix[6]arene skeleton. These catalysts can tune the selectivity of the catalytic cycloisomerization of 1,6-enynes in response to the relative orientation of the coordinated gold(I) atom with respect to the macrocycle.

One of the major challenges in organic synthetic chemistry is the control of reaction selectivity (site, chemo, stereo etc.). Site-reaction selectivity is always essential when multiple potential reactive sites are present in the substrate. Poor site-selectivity would result in complex and sometimes even unachievable separation and purification procedures. Rui Wang and Yang Yu [16] reported an interesting review in which they summarized various site-selective reactions mediated by molecular containers. They focused their attention on reactions that give different product distributions when performed inside the containers compared to the bulk solution.

As mentioned above, activation of substrates, stabilization of intermediates and transition states through intermolecular interactions was established as one of the fundamental factors of supramolecular catalysis, and among these hydrogen bonding interactions play a pivotal role in catalysis. More recently, halogen bonding interactions have been used as a novel tool to catalyze a wide variety of processes. Other nonclassical interactions, including anion-, chalcogen-, and pnictogen bonding, have also been exploited for the design of novel supramolecular catalysts.

In their contribution Wang and co-workers [17] reported an interesting example of chalcogen bonding catalysis approach for the synthesis of calix[4]pyrrole macrocycles. The Se···O=C chalcogen bonding interactions between a selenide-based catalyst and the carbonyl-substrate catalyzed the macrocyclization with pyrrole. Interestingly, only 5 mol % of catalyst loadings were necessary in order to promote the condensation processes, and calix[4]pyrrole derivatives were obtained in moderate to high yields. Mild reaction conditions were employed, thus highlighting the potential of this type of nonclassical interactions in catalyzing chemical transformations.

Among the examples of self-assembled catalytic capsules, the hexameric resorcinarene system was particularly investigated.

In their contribution, Alessandro Scarso and co-workers [18] showed that the hexameric capsule can catalyze the cyclization of (*S*)-citronellal forming isopulegol. In this study it was exploited the ability of the resorcinarene capsule to work as a Brønsted acid catalyst, and its aptitude to stabilize cationic intermediates and transition states inside the cavity.

Velmurugan, Hu and co-workers [19] reported an efficient photocatalytic supramolecular system based on a self-assembled nanosystem. The self-assembled system was obtained in an aqueous medium by inclusion of ammonium benzoyl-ʟ-alaninate (G) in a tetraphenylethylene-embedded pillar[5]arene (m-TPEWP5). The resulting worm-like supramolecular nanostructures, displayed aggregation-induced emission (AIE) due to the restricted phenyl-ring rotation of m-TPEWP5 component. Inspired by natural photosynthesis and following an energy transfer process, the supramolecular nanorod assembly was employed as a nanoreactor for a photocatalytic dehalogenation reaction, i.e., debromination of 2-bromo-1-phenylethanone derivatives, with high yields and short reaction times in an aqueous solution.

In the last decades, macrocyclic hosts have been widely used as supramolecular catalysts [7]. In this work, Qi-Qiang Wang [20] and co-workers reported tetraaminobisthiourea chiral macrocycles as catalysts in decarboxylative Mannich reactions. Low macrocycle loading was used to catalyze the decarboxylative addition of malonic acid half thioesters to isatin-derived ketimines with excellent yields and good enantioselectivity. It was reported that effective activation and stereocontrol of the reaction depends on the conformational rigidity of the macrocyclic framework and a synergy between the thiourea and tertiary amine sites.

Recently, many efforts were focused on the synthesis and applications of mechanically interlocked molecules (MIMs), such as catenanes and rotaxanes. MIMs show interesting structural and topological features and offer conceptually new possibilities as catalysts. In their minireview, Krajnc and Niemeyer [21] highlighted the use of the axially chiral 1,1'-binaphthyl-2,2'-diol (BINOL) unit as a stereogenic element in MIMs. The authors comment on the synthesis and properties of such BINOL-based chiral MIMs, together with their use in further diastereoselective modifications, their application in asymmetric catalysis, and stereoselective chemosensing.

In their minireview, Prodip Howlader and Michael Schmittel [22] highlighted the recent results in the field of the supramolecular catalysis on the use of discrete heteroleptic metallo-supramolecular complexes as catalysts. The idea of breaking/reducing symmetry has inspired many researchers to study heteroleptic metallo-complexes made up of various ligands. The authors emphasized the advantages of heteroleptic over homoleptic cages and they showed examples of nanomechanical motion influencing catalytic activity. They also discuss the regulation of the catalytic activity of heteroleptic systems by an external stimulus.

We are very grateful to all colleagues who contributed to this issue and to the Editorial Team of the Beilstein-Institut for their kind support. We are convinced that this thematic issue will stimulate new insights in the field of supramolecular catalysis.

Carmine Gaeta, Pablo Ballester, Qi-Qiang Wang

Salerno, Tarragona, Beijing, September 2022

## References

[1] Breslow R (1982). Science.

[2] Arnold F H (2019). Angew Chem, Int Ed.

[3] MacMillan D W C (2008). Nature.

[4] List B (2007). Chem Rev.

[5] Raynal M, Ballester P, Vidal-Ferran A, van Leeuwen P W N M (2014). Chem Soc Rev.

[6] Gaeta C, La Manna P, De Rosa M, Soriente A, Talotta C, Neri P (2021). ChemCatChem.

[7] Wang Q-Q, Liu Y, Chen Y, Zhang H-Y (2019). Supramolecular Catalysis Using Organic Macrocycles.

[8] Catti L, Zhang Q, Tiefenbacher K (2016). Chem – Eur J.

[9] Borsato G, Rebek J, Scarso A, Zecchina A, Bordiga S, Groppo E E (2011). Capsules and Cavitands: Synthetic Catalysts of Nanometric Dimension. From Selective Nanocatalysts and Nanoscience.

[10] Purse B W, Rebek J (2005). Proc Natl Acad Sci U S A.

[11] Toste F D (2018). Acc Chem Res.

[12] Yoshizawa M, Klosterman J K, Fujita M (2009). Angew Chem, Int Ed.

[13] Roberts D A, Pilgrim B S, Nitschke J R (2018). Chem Soc Rev.

[14] Brown C J, Toste F D, Bergman R G, Raymond K N (2015). Chem Rev.

[15] Giovanardi G, Secchi A, Arduini A, Cera G (2022). Beilstein J Org Chem.

[16] Wang R, Yu Y (2022). Beilstein J Org Chem.

[17] Tong Q, Zhao Z, Wang Y (2022). Beilstein J Org Chem.

[18] Lorenzetto T, Fabris F, Scarso A (2022). Beilstein J Org Chem.

[19] Bai Z, Velmurugan K, Tian X, Zuo M, Wang K, Hu X-Y (2022). Beilstein J Org Chem.

[20] Guo H, Ao Y-F, Wang D-X, Wang Q-Q (2022). Beilstein J Org Chem.

[21] Krajnc M, Niemeyer J (2022). Beilstein J Org Chem.

[22] Howlader P, Schmittel M (2022). Beilstein J Org Chem.

